# Highly Efficient Ag_3_PO_4_/g-C_3_N_4_ Z-Scheme Photocatalyst for Its Enhanced Photocatalytic Performance in Degradation of Rhodamine B and Phenol

**DOI:** 10.3390/molecules26072062

**Published:** 2021-04-03

**Authors:** Mingxi Zhang, Hanxiao Du, Juan Ji, Fengfeng Li, Y. C. Lin, Chenwei Qin, Ze Zhang, Yi Shen

**Affiliations:** 1 Light Alloy Research Institute, Central South University, Changsha 410083, China; zhangmingxi@csu.edu.cn; 2 Key Laboratory of Inorganic Nonmetallic Materials Hebei Province, College of Materials Science and Engineering, North China University of Science and Technology, Tangshan 063210, China; 13731002744@163.com (H.D.); j17732534673@163.com (J.J.); qincw64@hotmail.com (C.Q.); z17331243479@163.com (Z.Z.); shenyi@ncst.edu.cn (Y.S.); 3 School of Mechanical and Electrical Engineering, Central South University, Changsha 410083, China

**Keywords:** Ag_3_PO_4_, g-C_3_N_4_, semiconductor photocatalyst, Z-scheme mechanism

## Abstract

Ag_3_PO_4_/g-C_3_N_4_ heterojunctions, with different g-C_3_N_4_ dosages, were synthesized using an in situ deposition method, and the photocatalytic performance of g-C_3_N_4_/Ag_3_PO_4_ heterojunctions was studied under simulated sunlight conditions. The results revealed that Ag_3_PO_4_/g-C_3_N_4_ exhibited excellent photocatalytic degradation activity for rhodamine B (Rh B) and phenol under the same light conditions. When the dosage of g-C_3_N_4_ was 30%, the degradation rate of Rh B at 9 min and phenol at 30 min was found to be 99.4% and 97.3%, respectively. After five cycles of the degradation experiment for Rh B, g-C_3_N_4_/Ag_3_PO_4_ still demonstrated stable photodegradation characteristics. The significant improvement in the photocatalytic activity and stability of g-C_3_N_4_/Ag_3_PO_4_ was attributed to the rapid charge separation between g-C_3_N_4_ and Ag_3_PO_4_ during the Z-scheme charge transfer and recombination process.

## 1. Introduction

With the rapid development of industry, environmental pollution caused by industrial wastewater is becoming increasingly serious. Photocatalysis is an effective technology to degrade pollutants in water, which has been widely researched [1,2]. However, one-component semiconductor photocatalysts always face various defects, such as low visible-light availability and easy recombination of photogenerated charges. It has been proven that the construction of semiconductor heterostructures is an effective route to improve photocatalytic efficiency [3,4]. In recent years, an all-solid Z-scheme semiconductor composite photocatalyst has been applied in photocatalysis [5,6,7,8,9]. When Z-scheme photocatalysts are excited, h^+^ from the valence band (VB) at a higher energy level can combine with e^−^ from the conduction band (CB) at a lower energy level, while e^−^ with a stronger reducing ability in CB at a higher energy level and h^+^ with a stronger oxidation ability in lower VB at a lower energy level can participate in the reduction and oxidation processes during photocatalytic degradation, respectively. This method is conducive to obtain high charge separation efficiency and strong redox ability simultaneously, thus improving the photocatalytic efficiency [8,9].

In recent years, Z-scheme Ag_3_PO_4_-based photocatalysts with a high photocatalytic activity have been designed and applied in wastewater treatment and environmental control [10,11,12,13], including Ag_3_PO_4_/MoS_2_ [14], Bi_2_MoO_6_/Ag_3_PO_4_ [15], Ag_3_PO_4_/Bi_2_WO_6_ [16], Ag_3_PO_4_RGO/BiMoO_4_ [17], AgPO_4_/Ag/WO_3−x_ [18], and Ag_3_PO_4_/Pd/LaPO_4_ [19]. Lamellar g-C_3_N_4_ nanosheets possess high surface area, suitable band gap (2.7 eV), low cost, and good thermal and chemical stability, which has attracted extensive attention in the field of photocatalysis [20,21,22,23]. When g-C_3_N_4_ is combined with Ag_3_PO_4_, the resultant g-C_3_N_4_/Ag_3_PO_4_ photocatalyst is expected to show significantly enhanced photocatalytic activity.

Among the many types of pollutants, dyes and dangerous compounds are two main pollutants in industrial wastewater. Rh B and phenol are the typical substances of the two pollutants, respectively. Rh B is very harmful to human health. It can cause redness of skin and viscera, mild congestion of cerebral vascular, rupture of myocardial fiber, and other symptoms. Phenol has a strong corrosive effect on skin and mucous membrane, inhibiting the central nervous system and damaging the function of liver and kidney, etc. In addition, phenol is more difficult to degrade than other pollutants in water. Thus, they were chosen as the degradation object in photocatalytic experiments.

In this paper, we synthesized the Ag_3_PO_4_/g-C_3_N_4_ Z-scheme heterojunction photocatalyst using the in situ deposition method and evaluated the photocatalytic activity by the degradation experiment for Rh B and phenol. The influence of g-C_3_N_4_ and Ag_3_PO_4_ on photocatalytic activity was studied in detail and the probable photocatalytic mechanism of Ag_3_PO_4_/g-C_3_N_4_ was proposed.

## 2. Experimental Section

### 2.1. Sample Preparation

Preparation of g-C_3_N_4_: A typical calcination method was used to prepare g-C_3_N_4_. Briefly, 10 g urea powder was placed in an alumina crucible with a lid. The crucible was heated in air at a heating rate of 2 °C·min^−1^ to 550 °C and, then held at this temperature for 2 h to obtain g-C_3_N_4_. Subsequently, the bulk g-C_3_N_4_ was thermally exfoliated into g-C_3_N_4_ nanosheets by calcination at 600 °C for 2 h in air. The light yellow product was collected and ground using an agate mortar for subsequent use.

Synthesis of Ag_3_PO_4_/g-C_3_N_4_: Fifty milligrams of g-C_3_N_4_ nanosheets were dispersed in 80 mL of deionized water by ultrasonication. Silver ammonia solution (0.1 g·L^−1^) was dropped into the aqueous dispersion of g-C_3_N_4_ nanosheets and, then magnetically stirred for 1 h to fully adsorb Ag(NH_3_)^2+^ ions on the surface of g-C_3_N_4_ nanosheets. Then, the KH_2_PO_4_ solution (0.1 g·L^−1^) was dropped into the above mixture under magnetic agitation and the mixture continued to be stirred for 1 h. The final product was collected by centrifugation, washed with deionized water and ethanol thrice, and dried at 70 °C for 1 h. Finally, the product was collected and ground with an agate mortar for further use. According to the theoretical dosage of g-C_3_N_4__,_ the as-prepared samples were named Ag_3_PO_4_/g-C_3_N_4_-10 wt%, Ag_3_PO_4_/g-C_3_N_4_-20 wt%, Ag_3_PO_4_/g-C_3_N_4_-30 wt%, and Ag_3_PO_4_/g-C_3_N_4_-40 wt%. The actual dosage of g-C_3_N_4_ detected by EDS were 9.2 wt%, 16.3 wt%, 27.7 wt%, and 41.8 wt%, respectively. In addition, the simple physical mixture of Ag_3_PO_4_ and 30 wt% g-C_3_N_4_ was named the Ag_3_PO_4_/g-C_3_N4-30% mixture_._

### 2.2. Sample Characterization

The crystal structure was analyzed by a Bruker D8 X-ray diffractometer (XRD, Bruker, Germany), equipped with a Cu K_α_ irradiation light source (λ = 0.154 nm). The microstructure was observed using a Tecnai G2 F20 transmission electron microscopy (TEM, FEI, Hillsboro, OR, USA). Room-temperature transient photoluminescence (PL) spectra were recorded using an FLS1000 spectrometer (EI, UK). UV-vis diffuse reflectance spectra (UV-Vis, Hitachi, Tokyo, Japan) were measured by using a UH4150 UV-Vis near-infrared spectrophotometer. The photocurrent response was measured using a CHI 760E electrochemical workstation (Chenhua, Shanghai, China).

### 2.3. Photocatalytic Activity Test

The photocatalytic activity was evaluated by the pollutant degradation experiments at room temperature. A Polfilet xenon lamp (300 W) with a 320-nm filter was used as the light source. The spectra of the xenon lamp are shown in Figure S1 and detailed experimental devices are shown in Figure S2. The reaction solution consisted of 50 mL of rhodamine B (Rh B, 5 mg·L^−1^) or 50 mL of phenol (10 mg·L^−1^), and the photocatalyst was 0.03 g Ag_3_PO_4_, g-C_3_N_4_, or Ag_3_PO_4_/g-C_3_N_4_. The photocatalyst was weighed and added to the reaction solution, and the reaction solution was continuously stirred in the dark for 30 min to achieve an adsorption–desorption balance between the photocatalytic material and pollutant. Subsequently, the solution was irradiated by a full-wavelength Xenon lamp, and the absorbance of the supernatant was measured at certain intervals. In the cyclic experiments, the photocatalyst was separated from the reaction system after each degradation experiment, washed with ethanol and deionized water, and re-dispersed in the newly-prepared reaction solution to repeat the degradation experiment.

## 3. Results and Discussion

### 3.1. Structural Analysis and Microstructure

**Figure 1:**
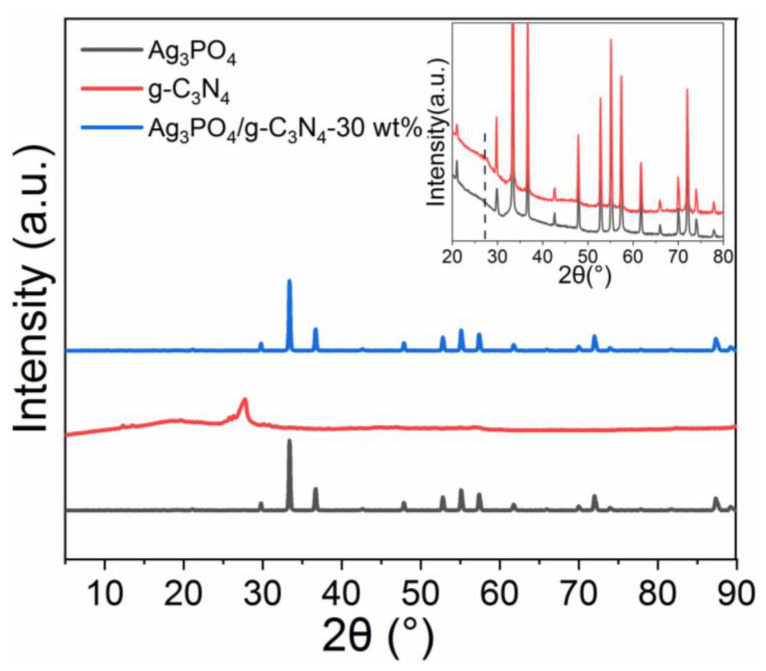
XRD patterns of as-prepared Ag_3_PO_4_, g-C_3_N_4_, and Ag_3_PO_4_/g-C_3_N_4._

Figure 1 shows the XRD patterns of Ag_3_PO_4_, g-C_3_N_4_ and Ag_3_PO_4_/g-C_3_N_4_-30 wt%. As shown in Figure 1, a strong peak appeared in the diffraction pattern of g-C_3_N_4_ at 2θ = 26.5°, corresponding to the (002) planes of g-C_3_N_4_ (JCPDS card no. 87-1526), which is the characteristic interlayer stacking peak of g-C_3_N_4_ [24]. The Ag_3_PO_4_ and Ag_3_PO_4_/g-C_3_N_4_-30 wt% exhibited similar XRD patterns and all strong diffraction peaks corresponded to the cubic Ag_3_PO_4_ phase (JCPDS card no. 06-0505). The inset provided the refined XRD patterns of Ag_3_PO_4_ and Ag_3_PO_4_/g-C_3_N_4_-30 wt%. Compared with Ag_3_PO_4_, the XRD pattern of Ag_3_PO_4_/g-C_3_N_4_ showed the characteristic peaks of g-C_3_N_4_; however, the peak intensities were far weaker than that of Ag_3_PO_4_. This may be attributed to the inferior crystallinity and lower content of well-exfoliated g-C_3_N_4_.

**Figure 2:**
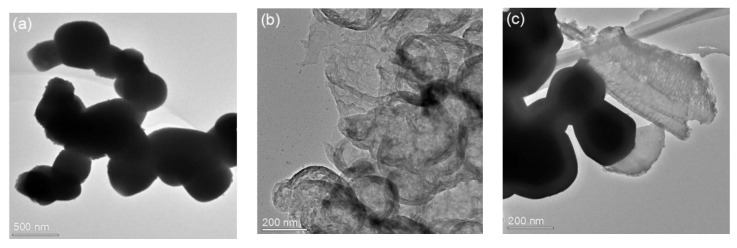
TEM images of (**a**) Ag_3_PO_4_, (**b**) g-C_3_N_4_, and (**c**) Ag_3_PO_4_/g-C_3_N_4._

Figure 2 shows TEM images of Ag_3_PO_4_, g-C_3_N_4_, and Ag_3_PO_4_/g-C_3_N_4_ photocatalysts. Figure 2a illustrates that Ag_3_PO_4_ consisted of approximately cubic particles with a size of 200–300 nm. As shown in Figure 2b, g-C_3_N_4_ presented thin wrinkled nanosheets. After thermal exfoliation, the specific surface area of g-C_3_N_4_ increased significantly, due to morphological changes. Figure 2c shows that the small-sized Ag_3_PO_4_ particles were attached to the surface of g-C_3_N_4_, forming a stable composite.

### 3.2. Optical Properties

**Figure 3:**
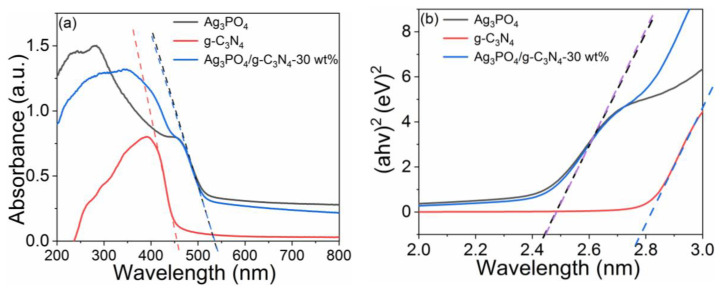
(**a**) UV-vis diffuse reflectance spectra, (**b**) estimated bandgap of Ag_3_PO_4_, g-C_3_N_4_, and Ag_3_PO_4_/g-C_3_N_4_-30 wt%.

Figure 3 shows the UV-vis diffuse reflectance spectra of Ag_3_PO_4_, g-C_3_N_4_, and Ag_3_PO_4_/g-C_3_N_4_-30 wt% photocatalysts. As shown in Figure 3a, the absorption cutoff edges of Ag_3_PO_4_ and g-C_3_N_4_ were located at about 460 and 530 nm, respectively. Compared with Ag_3_PO_4_, the absorption edge of Ag_3_PO_4_/g-C_3_N_4_-30 wt% was basically unchanged. Based on the UV-vis absorption data, the bandgap width of the photocatalysts was calculated and results are shown in Figure 3b. The calculated bandgap width of g-C_3_N_4_ was about 2.78 eV, whereas the bandgap of Ag_3_PO_4_ and Ag_3_PO_4_/g-C_3_N_4_-30wt% decreased to 2.45 eV.

**Figure 4:**
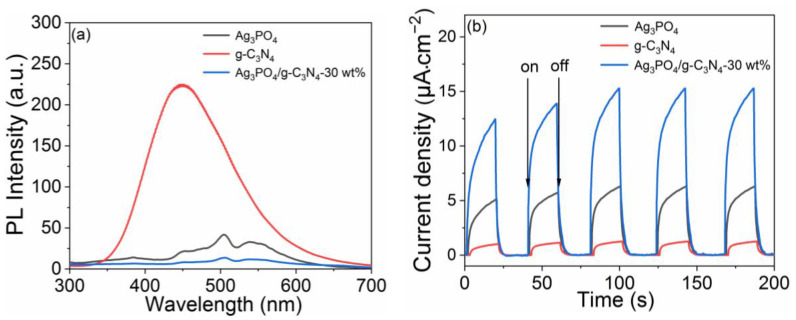
(**a**) Photoluminescence spectra and (**b**) transient photocurrent response curves of Ag_3_PO_4_, g-C_3_N_4_, and Ag_3_PO_4_/g-C_3_N_4_-30 wt%.

By testing the photoelectrochemical properties of Ag_3_PO_4_, g-C_3_N_4_, and Ag_3_PO_4_/g-C_3_N_4_-30 wt% photocatalysts, the separation and transfer efficiency of photogenerated electron-hole pairs were studied and results are shown in Figure 4. Figure 4a presents the photoluminescence (PL) spectra of the as-synthesized photocatalysts. The PL emission peak of g-C_3_N_4_ was located at 460 nm, showing the highest PL intensity and indicating that the photogenerated charge of g-C_3_N_4_ exhibited high recombination efficiency. The PL emission peak of Ag_3_PO_4_ was located at 460 nm, showing a far lower PL intensity than g-C_3_N_4_. When Ag_3_PO_4_ was combined with g-C_3_N_4_, the location of the PL emission peak of Ag_3_PO_4_/g-C_3_N_4_-30 wt% was basically the same as Ag_3_PO_4_, but the PL peak intensity of Ag_3_PO_4_/g-C_3_N_4_-30 wt% was significantly lower than Ag_3_PO_4_. Among Ag_3_PO_4_, g-C_3_N_4_, and Ag_3_PO_4_/g-C_3_N_4_-30 wt%, Ag_3_PO_4_/g-C_3_N_4_ exhibited the lowest PL peak intensity, which corresponded to the lowest recombination efficiency for photogenerated charges. As can be observed in Figure 4b, all photocatalyst electrodes exhibited rapid response when irradiated by a Xenon lamp (full wavelength). The Ag_3_PO_4_/g-C_3_N_4_-30 wt% showed the highest photocurrent response of about 16.35 μA·cm^−2^, which was 2.79 times higher than Ag_3_PO_4_ (5.87 μA·cm^−2^) and 21.8 times higher than g-C_3_N_4_ (0.75 μA·cm^−2^). These results indicate that the combination of Ag_3_PO_4_ and g-C_3_N_4_ reduced the recombination efficiency of photogenerated electrons and holes, and accelerated the charges transfer, which is beneficial for photocatalysis.

### 3.3. Photocatalytic Activity

**Figure 5:**
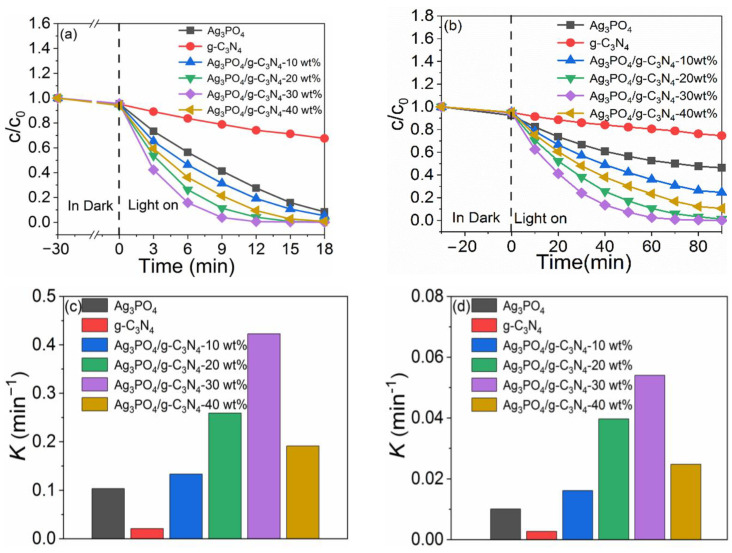
(**a**,**b**) Photocatalytic curves, (**c**,**d**) rate constants in the degradation of Rh B and phenol, with different g-C_3_N_4_ content.

Furthermore, using Rh B and phenol as target pollutants, we simulated the photocatalytic reaction under sunlight irradiation using Xenon lamp (full wavelength) irradiation, and evaluated the photocatalytic activity, as shown in Figure 5. Figure 5a shows the photocatalytic activity of Ag_3_PO_4_/g-C_3_N_4_ with different amounts of g-C_3_N_4_ After irradiation by the Xenon lamp for 9 min, the photocatalytic degradation rate of RhB by Ag_3_PO_4_, g-C_3_N_4_, Ag_3_PO_4_/g-C_3_N_4_-10 wt%, Ag_3_PO_4_/g-C_3_N_4_-20 wt%, Ag_3_PO_4_/g-C_3_N_4_-30 wt%, and Ag_3_PO_4_/g-C_3_N_4_-40 wt% was found to be 71.1%, 22.2%, 79.8%, 95.5%, 99.4%, and 89.9%, respectively. With the increase of g-C_3_N_4_ content, the photocatalytic activity of Ag_3_PO_4_/g-C_3_N_4_ initially increased, followed by a decrease. The optimal photocatalytic activity was achieved for Ag_3_PO_4_/g-C_3_N_4_-30 wt%. The first-order kinetic model [25,26] was used to calculate the corresponding reaction rate constants (*k*), and the results are shown in Figure 5c. The observed reaction rate constant of Ag_3_PO_4_, g-C_3_N_4_, Ag_3_PO_4_/g-C_3_N_4_-10 wt%, Ag_3_PO_4_/g-C_3_N_4_-20 wt%, Ag_3_PO_4_/g-C_3_N_4_-30 wt%, and Ag_3_PO_4_/g-C_3_N_4_-40 wt% was found to be 0.1033, 0.0209, 0.1333, 0.2591, 0.4227, and 0.1911 min^−1^, respectively. The *k* value of Ag_3_PO_4_/g-C_3_N_4_-30 wt% (0.4227 min^−1^) was the highest, which was ≈4.09 and 20.24 times higher than Ag_3_PO_4_ and g-C_3_N_4_, respectively.

In order to further verify the superior photocatalytic activity of Ag_3_PO_4_/g-C_3_N_4_, the photocatalytic degradation experiment for phenol was also carried out and the results are shown in Figure 5b. Under Xenon lamp irradiation for 30 min, the degradation rate of phenol by Ag_3_PO_4_, g-C_3_N_4_, Ag_3_PO_4_/g-C_3_N_4_-10 wt%, Ag_3_PO_4_/g-C_3_N_4_-20 wt%, Ag_3_PO_4_/g-C_3_N_4_-30 wt%, and Ag_3_PO_4_/g-C_3_N_4_-40 wt% was found to be 43.0%, 15.8%, 63.9%, 90.9%, 99.6%, and 77.5%, respectively. Figure 5d shows that the Ag_3_PO_4_/g-C_3_N_4_-30 wt% exhibits the highest rate constant *k* (0.0540 min^−1^), which was ≈5.35 and 20.00 times higher than Ag_3_PO_4_ (0.01009 min^−1^) and g-C_3_N_4_ (0.0027 min^−1^), respectively. Hence, Ag_3_PO_4_/g-C_3_N_4_ showed obvious advantages for the degradation of pollutants.

**Figure 6:**
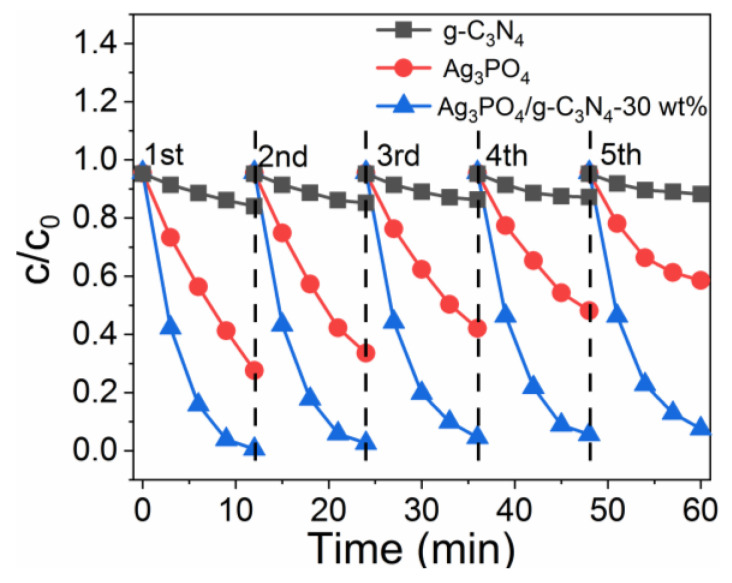
Recycling runs results of Ag_3_PO_4_, g-C_3_N_4_, and Ag_3_PO_4_/g-C_3_N_4_-30 wt% in degradation of Rh B .

Figure 6 presents the cyclic stability of Rh B degradation by Ag_3_PO_4_, g-C_3_N_4_, and Ag_3_PO_4_/g-C_3_N_4_-30 wt% photocatalysts. Under Xenon lamp irradiation, the loss rate of Rh B degradation by Ag_3_PO_4_, g-C_3_N_4_, and Ag_3_PO_4_/g-C_3_N_4_-30 wt% during the fifth cycle, compared with the initial degradation, was 32.5%, 11.5%, and 7.3%, respectively. The presence of g-C_3_N_4_ significantly reduced the loss rate for Rh B and phenol degradation. Hence, Ag_3_PO_4_/g-C_3_N_4_ showed excellent photocatalytic stability.

### 3.4. Photocatalysis Species

**Figure 7:**
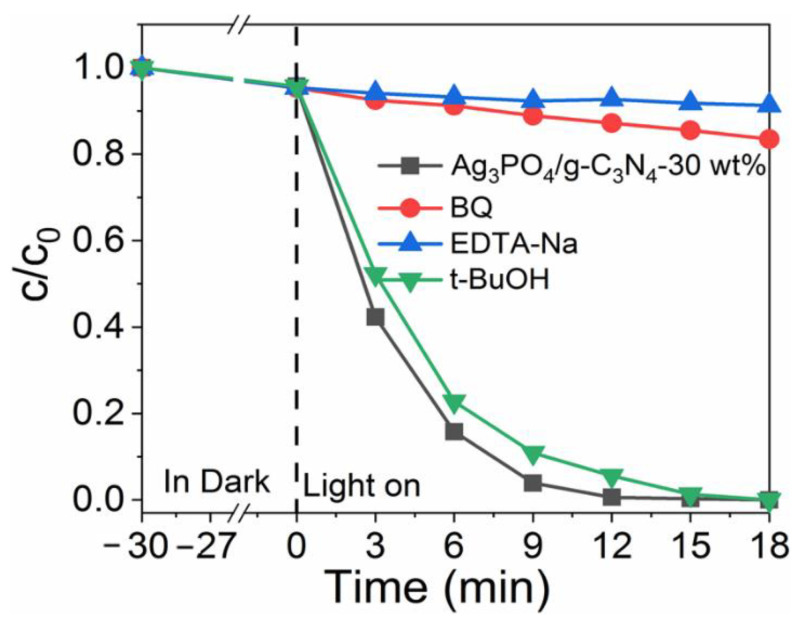
Photocatalytic activities of Ag_3_PO_4_/g-C_3_N_4_-30wt% for the degradation of Rh B in the presence of different scavengers.

In order to identify the active species during the photocatalytic process, free radical capture experiments were carried out using Rh B as a target pollutant. EDTA-2Na, p-benzoquinone (BZQ), and tert-butanol were introduced during the photocatalytic process as h^+^, ·O_2_^−^, and OH^−^ inhibitors, respectively, and the results are shown in Figure 7. The introduction of tert-butanol during the photocatalytic process of Ag_3_PO_4_/g-C_3_N_4_-30 wt% rendered no influence on the photodegradation efficiency of Rh B, whereas EDTA-2Na and BZQ both significantly reduced the degradation efficiency of Rh B with a degradation rate of 4.4% and 12.4%, respectively. These results indicate that h^+^ and O^2−^ are the main active species in Ag_3_PO_4_/g-C_3_N_4-_30 wt%.

### 3.5. Energy Band Structure and Photocatalytic Mechanism

**Figure 8:**
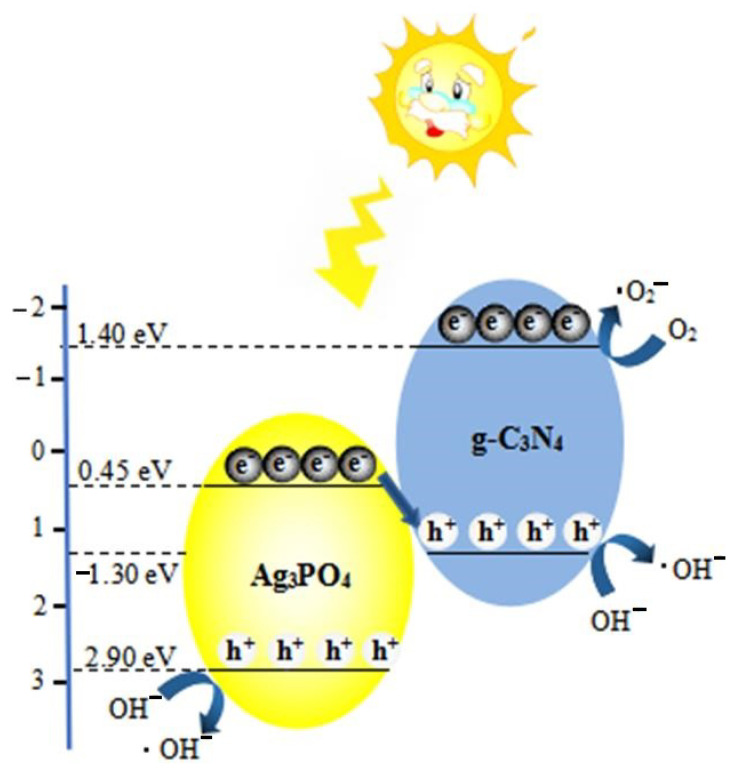
Energy band structure and Z-scheme photocatalytic mechanism of Ag_3_PO_4_/g-C_3_N_4._

Figure 8 presents the Z-scheme charge transfer pathway of the Ag_3_PO_4_/g-C_3_N_4_ composite photocatalyst for the degradation of organic pollutants. The bandgap of g-C_3_N_4_ was 2.7 eV with the VB potential of ~ 1.4 eV and CB potential of ~ −1.3 eV [27,28]. The potential of e^−^ on the CB of g-C_3_N_4_ was −1.3 eV, which can reduce the molecular oxygen O_2_ to·O^2^ because the potential of O_2_/·O_2_^–^ was −0.44 eV vs. NHE. Therefore, O^2−^ was the main active substance during the photocatalytic process by g-C_3_N_4_. The bandgap of Ag_3_PO_4_ was 2.45 eV with a VB potential of ~2.9 eV and CB potential of ~0.45 eV [29]. The generated electrons (e^−^) in the CB of Ag_3_PO_4_ are insufficient to reduce O_2_ into O^2−^. Therefore, holes (h^+^) play a major role during the photocatalytic degradation of organic matter by Ag_3_PO_4_.

Based on the energy band analysis, it can be inferred that the photogenerated e^−^ in the CB of Ag_3_PO_4_ can combine with h^+^ in the VB of g-C_3_N_4_ due to the formation of a heterojunction interface between Ag_3_PO_4_ particles and g-C_3_N_4_ nanosheets, resulting in the accumulation of e^−^ in the CB of g-C_3_N_4_ and h^+^ in VB of Ag_3_PO_4_. The h^+^ in the VB of Ag_3_PO_4_ can directly react with pollutants, whereas the electrons in CB of g-C_3_N_4_ can reduce O_2_ into O^2−^, which reacts with pollutants. The Z-scheme charge transfer mechanism promotes the separation of electron-hole pairs, slows down the photocorrosion of Ag^+^, and improves photocatalyst activity and stability.

## 4. Conclusions

In summary, the Z-scheme heterojunction Ag_3_PO_4_/g-C_3_N_4_ photocatalyst was synthesized using an in situ deposition method and exhibited excellent photocatalytic degradation activity for Rh B and phenol under Xenon lamp irradiation. The observed rate constant (*k*) for the degradation of Rh B by Ag_3_PO_4_/g-C_3_N_4_ was found to be 0.4227 min^−1^, which was 4.09 and 20.24 times higher than pure Ag_3_PO_4_ and g-C_3_N_4_, respectively. Moreover, the *k* value for the degradation of phenol by Ag_3_PO_4_/g-C_3_N_4_ was 0.0540 min^−1^, which was 5.35 and 20.00 times higher than pure Ag_3_PO_4_ and g-C_3_N_4_, respectively. Overall, the formation of the Z-scheme heterojunction hindered the recombination of photogenerated electrons and holes, and accelerated the electron transfer, thus improving the activity and stability of photocatalysts.

## Data Availability

The data presented in this study are available on request from the corresponding author. The data are not publicly available due to the ongoing follow-up studies.

## Supplementary Materials

Figure S1: The spectra of xenon lamp, Figure S2: The picture of experimental setup.

## Sample Availability

Samples of the Ag_3_PO_4_/g-C_3_N_4_.photocatalyst are available from the authors. However, it may be necessary to pay properly for the synthesis and mailing of samples.
