# Supplementary material for: Highly Efficient Ag3PO4/g-C3N4 Z-Scheme Photocatalyst for Its Enhanced Photocatalytic Performance in Degradation of Rhodamine B and Phenol
Source: Molecules. 2021 Apr 3;26(7):2062. doi: 10.3390/molecules26072062 (PMC8038389; doi:10.3390/molecules26072062)
Supplement: Supplementary file 1 [file molecules-26-02062-s001.pdf]

Supplementary Materials

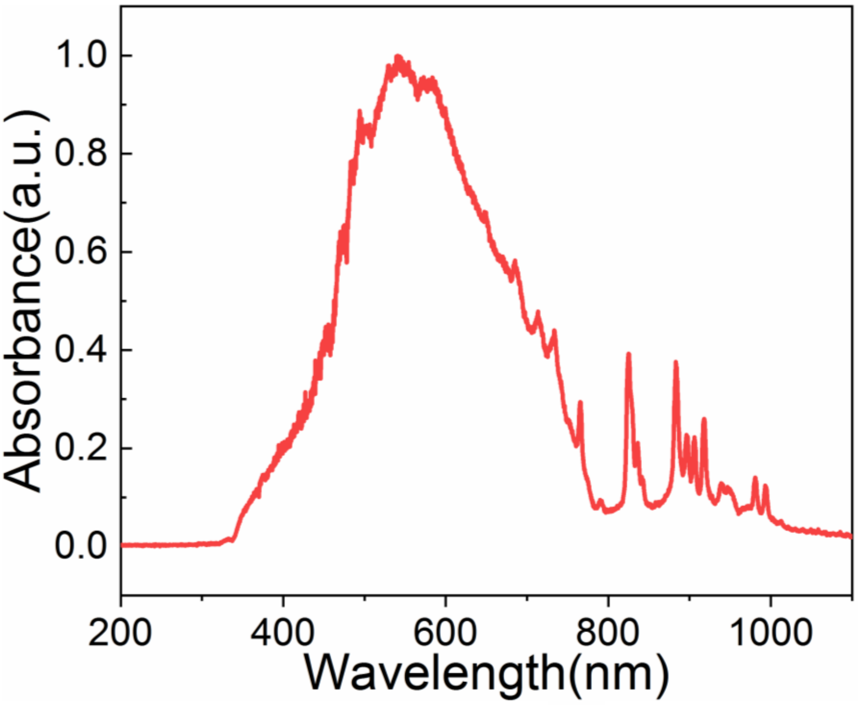

Figure S1 The spectra of xenon lamp.

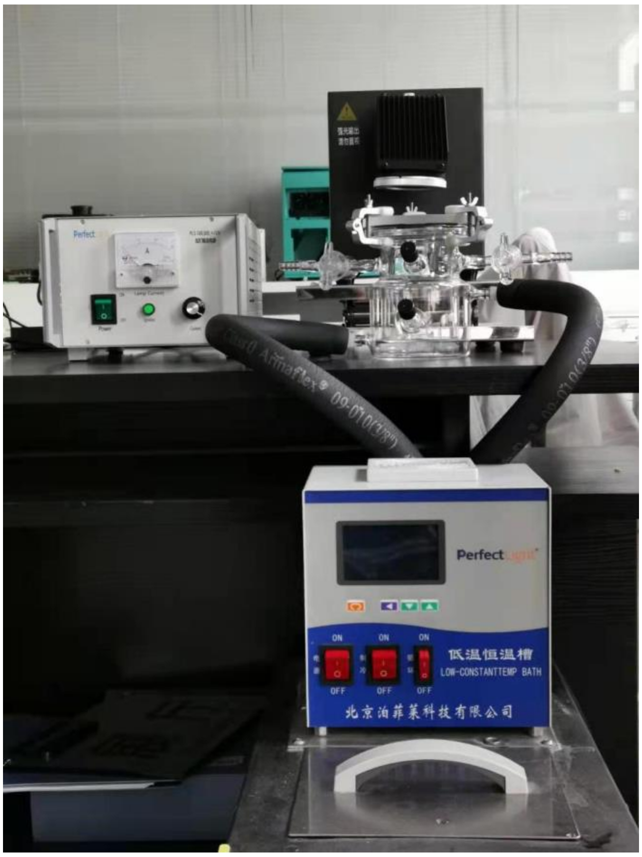

Figure S2 The picture of experimental setup.
